# Supplementary material for: Trends and projections of universal health coverage indicators in Ghana, 1995-2030: A national and subnational study
Source: PLoS One. 2019 May 22;14(5):e0209126. doi: 10.1371/journal.pone.0209126 (PMC6530887; doi:10.1371/journal.pone.0209126)
Supplement: S1 Appendix — (DOCX) [file pone.0209126.s001.docx]

**Supplementary appendix**

**Trends and projections of universal health coverage indicators in Ghana, 1995-2030: A national and subnational study**

**Reference**

1. Boerma T, AbouZahr C, Evans D, Evans T. Monitoring Intervention Coverage in the Context of Universal Health Coverage. *PLoS Med* 2014;**11**: e1001728
2. Boerma JT, Bryce J, Kinfu Y, Axelson H, Victora CG. Mind the gap:equity and trends in coverage of maternal, newborn, and child health services in 54 countdown countries. *Lancet* 2008, **371**:1259–67.
3. Ghana Statistical Services. Ghana Living Standards Survey Report on the Third Round (GLSS 3). Ghana, 1995.
4. Ghana Statistical Services, National Data Archive. Ghana Living Standard Survey-1998, With labour force model. Ghana, 2000.
5. Ghana Statistical Services, National Data Archive. Ghana Living Standard Survey 5: 2005, With Non-Farm Household Enterprise Module. Ghana, 2008.
6. Ghana Statistical Services, Macro International Inc. Ghana Demographic and Health Survey 1993. Calverton, Maryland, USA, 1994.
7. Ghana Statistical Services, Macro International Inc. Ghana Demographic and Health Survey 1998. Calverton, Maryland, 1999.
8. Ghana Statistical Services, Noguchi Memorial Institute for Medical Research, ORC Macro. Ghana Demographic and Health Survey 2003. Calverton, Maryland, 2004.
9. Ghana Statistical Services, Ghana Health Service, ICF International. Ghana Demographic and Health Survey 2014. Rockville, Maryland, 2015.
10. Ghana Statistical Services, Ghana Health Service, ICF Macro. Ghana Demographic and Health Survey 2008. Calverton, Maryland, 2009.
11. Ghana Stastitical Services, National Data Archive. Ghana Living Standards Survey 6 (With a Labour Force Module) 2012-2013, Round Six. Ghana, 2014.
12. World Health Organization. Tracking universal health coverage: first global monitoring report*.* Geneva: World Health Organization, 2015.
