# Supplementary material for: Trends and projections of universal health coverage indicators in Ghana, 1995-2030: A national and subnational study
Source: PLoS One. 2019 May 22;14(5):e0209126. doi: 10.1371/journal.pone.0209126 (PMC6530887; doi:10.1371/journal.pone.0209126)
Supplement: S1 Table — (DOCX) [file pone.0209126.s002.docx]

**S1 Table: Health service indicators**

| Indicator | Abbreviation | | Definition^a^ |
| --- | --- | --- | --- |
| Prevention indicators | | | |
| At least four antenatal care visits | ANC4+ | The proportion of women aged 15-49 years in the three years preceding the survey who received at least four visits from a skilled health provider (doctor, nurse or midwife) during their last pregnancy | |
| Post-natal care of mother | PNC | The proportion of women giving birth in the three years preceding the survey who received their first post-natal checkup in the first two days after birth from a medically trained provider (doctor, nurse, midwife, community skilled birth attendant) | |
| BCG immunization | BCG | The proportion of children aged 12-23 months who received one dose of Bacille Calmette-Guerin (BCG) vaccine | |
| DPT3 immunization | DPT3 | The proportion of children aged 12-23 months who received three doses of diphtheria, pertussis, and tetanus vaccine | |
| Polio3 immunization | Polio3 | The proportion of children aged 12-23 months who received three doses of polio vaccine | |
| Measles immunization | MSL | The proportion of children aged 12-23 months currently vaccinated against measles | |
| Exclusive breastfeeding | EBF | The proportion of the youngest child under two years of age living with the mother who are exclusively breastfed (based on a 24-hour recall period) | |
| Needs for family planning satisfied | FPS | The proportion of currently married women aged 15-49 who do not want any more children or want to wait two or more years before having another child and are using contraception | |
| Non-use of tobacco | NTobacco | The proportion of males aged 15-49 years old who do not use tobacco | |
| Insecticide treated bed nets for children | ITNC | Children under 5 who slept under an insecticide-treated bed net the previous night among all households | |
| Insecticide treated bed nets for pregnant women | ITNW | Pregnant women aged 15-49 who slept under an insecticide-treated bed net the previous night among all households | |
| Improved water | ImpWater | The proportion of households whose main source of drinking water is an improved source | |
| Adequate sanitation | Sanitation | The proportion of households with improved toilet facilities (flush to piped sewer system, flush to sewer system, flush to septic tank, and ventilated improved pit latrine) | |
| Treatment indicators | |  | |
| Institutional delivery | INSD | The proportion of live births delivered at a health facility in the three years preceding the survey | |
| Skilled birth attendance | SBA | The proportion of live births assisted by a skilled health provider (doctor, nurse, and midwife) in the three years preceding the survey | |
| Oral rehydration therapy for diarrheal treatment | ORT | The proportion of children aged under 5 years with diarrhea who received oral rehydration therapy (oral rehydration salts, recommended home solution, or increased fluids) in the previous two weeks | |
| Care seeking for pneumonia | CPNM | The proportion of children under 5 with suspected pneumonia who sought care from an appropriate health provider in the previous two weeks | |

Note: ^a^The definitions of the indicators were adapted from the global monitoring report of UHC^1^ and Boerma et al^2,3^
