# Supplementary material for: Trends and projections of universal health coverage indicators in Ghana, 1995-2030: A national and subnational study
Source: PLoS One. 2019 May 22;14(5):e0209126. doi: 10.1371/journal.pone.0209126 (PMC6530887; doi:10.1371/journal.pone.0209126)
Supplement: S2 Table — (DOCX) [file pone.0209126.s003.docx]

**S2 Table: Deviance information criteria for health service indicators**

| **Indicators** | **Model without interaction** | | | | | | | | **Model with interaction** | | | | | | | | | **Interaction used in the model** |
| --- | --- | --- | --- | --- | --- | --- | --- | --- | --- | --- | --- | --- | --- | --- | --- | --- | --- | --- |
|  | **Mean deviance** | | **Penalty** | | **Penalized deviance** | | | **Mean deviance** | | | **Penalty** | | | **Penalized deviance** | | | |  |
| **Quintile-specific** | |  | | | |  | | |  | | |  | | |  | | |  |
| FPS | -1.0 | | 19.9 | | 19.0 | | | -10.0 | | | 28.4 | | | 18.5 | | | yes | |
| ANC4+ | -14.3 | | 6.0 | | -8.2 | | | 33.0 | | | 13.0 | | | 46.0 | | | no | |
| PNC | 61.9 | | 8.3 | | 70.3 | | | 52.2 | | | 14.0 | | | 66.2 | | | yes | |
| EBF | 67.8 | | 5.3 | | 73.0 | | | 69.4 | | | 11.2 | | | 80.6 | | | no | |
| ITNC | 48.8 | | 8.9 | | 57.7 | | | 39.4 | | | 6.8 | | | 46.2 | | | yes | |
| ITNW | 35.5 | | 3.1 | | 37.7 | | | 34.7 | | | 14.4 | | | 49.1 | | | no | |
| BCG | 21.6 | | 6.0 | | 27.7 | | | 18.4 | | | 12.5 | | | 31.0 | | | no | |
| DPT3 | 28.2 | | 4.5 | | 32.7 | | | 31.5 | | | 18.0 | | | 49.5 | | | no | |
| Polio3 | 29.5 | | 4.0 | | 33.5 | | | 14.2 | | | 8.0 | | | 22.2 | | | yes | |
| MSL | 27.3 | | 13.0 | | 40.3 | | | 28.3 | | | 17.2 | | | 45.6 | | | no | |
| Impwater | 67.9 | | 8.3 | | 76.2 | | | 69.0 | | | 9.6 | | | 78.6 | | | no | |
| Sanitation | 45.9 | | 5.0 | | 50.9 | | | 49.3 | | | 8.8 | | | 58.1 | | | no | |
| Ntobacco | 3.8 | | 2.7 | | 6.5 | | | 1.5 | | | 12.1 | | | 13.6 | | | no | |
| INSD | 23.2 | | 11.4 | | 34.6 | | | 29.3 | | | 18.9 | | | 48.2 | | | no | |
| SBA | 29.2 | | 10.6 | | 39.8 | | | 20.7 | | | 16.4 | | | 37.1 | | | yes | |
| ORT | 35.8 | | 4.8 | | 40.6 | | | 36.6 | | | 6.9 | | | 43.4 | | | no | |
| CPNM | 38.9 | | 7.5 | | 46.4 | | | 31.7 | | | 4.6 | | | 36.3 | | | yes | |
| Prevention index | -10.4 | | 4.09 | | -6.36 | | | -18.5 | | | 22.5 | | | 3.9 | | | no | |
| Treatment index | -16.4 | | 5.2 | | -11.2 | | | -18.3 | | | 18.3 | | | 0.0 | | | no | |
| CCI | -23.0 | | 8.4 | | -14.6 | | | 55.1 | | | 8.1 | | | -47.0 | | | yes | |
| **Subnational** |  | | |  | | |  | | |  | | |  | | |  | | |
| Prevention index | 3.9 | | 8.2 | | 12.2 | | | 8.7 | | | 14.1 | | | 22.7 | | | no | |
| Treatment index | 20.1 | | 14.2 | | 32.2 | | | 7.1 | | | 23.5 | | | 30.6 | | | yes | |

Note: ANC4+: at least four antenatal care visits; PNC: post-natal care of mother; BCG: BCG immunization; DPT3: three doses of DPT immunization; Polio3: three doses of polio immunization; MSL: measles vaccination; EBF: exclusive breastfeeding; FPS: family planning needs satisfied; NTobacco: non-use of tobacco; Impwater: improved water; Sanitation: adequate sanitation; INSD: institutional delivery; SBA: skilled birth attendance; ORT: oral rehydration therapy for diarrheal treatment; CPNM: care seeking for pneumonia; ITNC: children under 5 who slept under an insecticide-treated bed net; ITNW: pregnant women who slept under an insecticide-treated bed net.
