# Supplementary material for: Trends and projections of universal health coverage indicators in Ghana, 1995-2030: A national and subnational study
Source: PLoS One. 2019 May 22;14(5):e0209126. doi: 10.1371/journal.pone.0209126 (PMC6530887; doi:10.1371/journal.pone.0209126)
Supplement: S3 Table — (DOCX) [file pone.0209126.s004.docx]

**S3 Table: Quintile-specific coverage of reproductive, maternal, and child health services in Ghana, 1995-2030**

| **Indicators** | **Predicted coverage in year (95% CrI)** | | | | | **Probability^a^** |
| --- | --- | --- | --- | --- | --- | --- |
|  | **1995** | **2005** | **2015** | **2030** | |  |
| **FPS** |  |  |  |  | |  |
| Poorest | 21.8 (27.8-26.3) | 29.2 (25.0-33.1) | 38.1 (31.1-45.1) | 52.6 (37.6-66.5) | | 0% |
| Poorer | 25.8 (21.2-30.4) | 35.0 (31.4-38.7) | 45.4 (38.0-52.8) | 61.4 (46.8-74.3) | | 0% |
| Middle class | 33.3 (28.0-38.5) | 38.8 (35.1-42.6) | 44.6 (36.9-52.0) | 53.5 (37.7-67.3) | | 0% |
| Richer | 42.5 (36.7-48.8) | 45.8 (41.7-49.9) | 49.1 (41.5-56.7) | 54.1 (38.4-67.4) | | 0% |
| Richest | 56.2 (50.1-62.7) | 55.5 (51.3-59.5) | 54.7 (46.8-62.2) | 53.5 (38.0-67.9) | | 0% |
| **ANC4+** |  |  |  |  | |  |
| Poorest | 38.8 (34.0-43.6) | 58.3 (54.0-62.5) | 75.6 (71.1-79.5) | 91.0 (87.6-93.4) | | 100% |
| Poorer | 48.0 (42.8-52.9) | 67.1 (63.1-70.8) | 81.8 (78.4-84.7) | 93.6 (91.3-95.4) | | 100% |
| Middle class | 55.2 (50.3-60.0) | 73.2 (69.5-76.6) | 85.7 (82.8-88.2) | 95.2 (93.3-96.6) | | 100% |
| Richer | 72.0 (68.2-75.9) | 85.0 (82.7-87.3) | 92.6 (91.1-94.1) | 97.6 (96.7-98.3) | | 100% |
| Richest | 86.1 (83.5-88.4) | 93.2 (91.9-94.2) | 96.8 (96.1-97.4) | 99.0 (98.6-99.3) | | 100% |
| **PNC** |  |  |  |  | |  |
| Poorest | ­0.7 (0.1-3.2) | 7.8 (3.2-15.4) | 59.0 (31.1-83.8) | 97.4 (81.7-100.0) | | 97.9% |
| Poorer | 0.3 (0.0-1.3) | 8.1 (3.3-15.9) | 76.8 (51.1-92.7) | 99.7 (97.6-100.0) | | 100% |
| Middle class | 0.7 (0.0-3.2) | 13.7 (5.8-25.4) | 83.4 (64.0-95.0) | 99.7 (98.2-100.0) | | 99.9% |
| Richer | 0.0 (0.0-0.2) | 8.4 (3.5-16.9) | 96.9 (91.6-99.2) | 100.0 (99.9-100.0) | | 100% |
| Richest | 0.1 (0.0-0.6) | 16.8 (7.4-32.2) | 97.9 (94.0-99.5) | 100.0 (100.0-100.0) | 100% | |
| **EBF** |  |  |  |  |  | |
| Poorest | 16.1 (9.3-24.7) | 39.2 (27.1-51.4) | 68.2 (53.5-80.7) | 92.5 (83.5-97.3) | 99.2% | |
| Poorer | 13.9 (8.0-22.1) | 35.1 (24.2-48.0) | 64.4 (49.8-78.2) | 91.3 (81.5-97.0) | 98.2% | |
| Middle class | 15.8 (9.2-24.5) | 38.5 (27.1-51.3) | 67.6 (52.5-80.3) | 92.4 (83.3-97.4) | 99.0% | |
| Richer | 19.2 (11.2-29.0) | 44.2 (30.9-57.1) | 72.5 (57.4-84.4) | 93.8 (85.7-98.0) | 99.6% | |
| Richest | 23.7 (14.6-35.4) | 51.0 (38.1-64.4) | 77.6 (65.0-87.7) | 95.2 (89.2-98.5) | 100% | |

Note: ^a^The probability of meeting the target of 80% health service coverage by 2030; CrI: credible interval; ANC4+: at least four antenatal care visits; PNC: postnatal care of mother; EBF: exclusive breastfeeding; FPS, family planning needs satisfied; ITNC: children under 5 who slept under an insecticide-treated bed net; ITNW: pregnant women who slept under an insecticide treated bed net. ^a^The probability of meeting the target of 80% health service coverage by 2030
