# Supplementary material for: Trends and projections of universal health coverage indicators in Ghana, 1995-2030: A national and subnational study
Source: PLoS One. 2019 May 22;14(5):e0209126. doi: 10.1371/journal.pone.0209126 (PMC6530887; doi:10.1371/journal.pone.0209126)
Supplement: S4 Table — (DOCX) [file pone.0209126.s005.docx]

**S4 Table: Quintile-specific vaccination coverage in Ghana, 1995-2030**

| Indicators | Predicted coverage in year (95% CrI) | | | | Probability^a^ |
| --- | --- | --- | --- | --- | --- |
|  | **1995** | **2005** | **2015** | **2030** |  |
| BCG immunization | |  |  |  |  |
| Poorest | 71.7 (60.5-80.6) | 88.2 (84.5-91.4) | 95.5 (92.1-97.7) | 98.9 (96.9-99.7) | 100% |
| Poorer | 82.6 (75.0-885) | 92.1 (89.4-94.2) | 96.5 (94.1-98.1) | 98.9 (97.1-99.7) | 100% |
| Middle class | 88.5 (83.2-92.7) | 95.4 (93.8-96.6) | 98.1 (96.7-99.0) | 99.5 (98.7-99.9) | 100% |
| Richer | 89.8 (84.9-93.6) | 94.5 (92.4-96.2) | 97.0 (94.4-98.6) | 98.7 (95.8-99.7) | 100% |
| Richest | 94.8 (92.1-96.8) | 97.6 (96.7-98.3) | 98.9 (98.1-99.4) | 99.6 (99.0-99.9) | 100% |
| DPT3 immunization | |  |  |  |  |
| Poorest | 57.6 (47.6-67.1) | 74.5 (66.5-80.7) | 86.2 (79.7-90.8) | 95.0 (90.7-97.5) | 100% |
| Poorer | 65.8 (56.5-74.1) | 80.5 (74.4-85.4) | 89.8 (85.3-93.4) | 96.4 (93.7-98.3) | 100% |
| Middle class | 66.9 (58.1-74.7) | 81.3 (75.1-86.5) | 90.3 (85.1-93.7) | 96.6 (93.7-98.4) | 100% |
| Richer | 75.4 (67.0-82.0) | 86.9 (82.1-90.5) | 93.4 (90.2-95.6) | 97.7 (95.8-98.9) | 100% |
| Richest | 82.7 (76.9-87.9) | 91.2 (88.2-93.8) | 95.7 (93.6-97.2) | 98.5 (97.2-99.3) | 100% |
| Polio3 immunization | |  |  |  |  |
| Poorest | 51.6 (41.2-62.9) | 74.6 (68.2-80.4) | 88.8 (82.0-93.5) | 97.0 (92.0-99.2) | 100% |
| Poorer | 65.1 (53.6-74.5) | 79.1 (73.7-83.5) | 88.2 (81.2-93.2) | 95.1 (87.5-98.7) | 100% |
| Middle class | 68.0 (58.0-77.7) | 79.0 (73.4-83.8) | 86.7 (79.0-92.2) | 93.2 (82.7-98.1) | 98.9% |
| Richer | 73.9 (64.8-82.1) | 83.1 (78.4-87.1) | 89.2 (82.8-93.8) | 94.3 (86.0-98.4) | 99.3% |
| Richest | 85.6 (78.7-90.7) | 85.5 (80.9-89.0) | 85.0 (76.4-91.3) | 83.4 (63.0-95.0) | 71.8% |
| Measles immunization | |  |  |  |  |
| Poorest | 59.5 (49.7-68.0) | 76.1 (69.2-81.8) | 87.3 (82.1-91.4) | 95.5 (92.2-97.7) | 100% |
| Poorer | 66.7 (56.8-75.3) | 81.2 (75.3-86.4) | 90.3 (86.0-93.7) | 96.7 (94.3-98.3) | 100% |
| Middle class | 71.1 (62.9-78.4) | 84.2 (79.1-88.3) | 92.0 (88.5-94.7) | 97.3 (95.2-98.7) | 100% |
| Richer | 77.1 (69.5-83.6) | 88.0 (84.1-91.6) | 94.0 (91.3-96.3) | 98.0 (96.5-99.0) | 100% |
| Richest | 84.4 (79.0-88.9) | 92.2 (89.2-94.4) | 96.2 (94.3-97.6) | 98.7 (97.7-99.4) | 100% |

Note: ^a^The probability of meeting the target of 80% health service coverage by 2030; CrI: credible interval; DPT3: three doses of DPT immunization; Polio3: three doses of polio immunization
