# Supplementary material for: Trends and projections of universal health coverage indicators in Ghana, 1995-2030: A national and subnational study
Source: PLoS One. 2019 May 22;14(5):e0209126. doi: 10.1371/journal.pone.0209126 (PMC6530887; doi:10.1371/journal.pone.0209126)
Supplement: S5 Table — (DOCX) [file pone.0209126.s006.docx]

**S5 Table: Quintile specific coverage of disease prevention and environmental health indicators in Ghana, 1995-2030**

| **Indicators** | **Predicted coverage in year (95% CrI)** | | | | |
| --- | --- | --- | --- | --- | --- |
|  | **1995** | **2005** | **2015** | **2030** | **Probability^a^** |
| **Non-communicable disease:** | |  |  |  |  |
| **Non-use of tobacco** | |  |  |  |  |
| Poorest | − | 81.0 (74.9-86.4) | 89.2 (84.3-92.8) | 95.5 (90.3-98.2) | 100% |
| Poorer | − | 90.0 (86.2-93.0) | 94.6 (91.8-96.5) | 97.8 (95.2-99.1) | 100% |
| Middle class | − | 92.3 (89.4-94.6) | 95.9 (93.9-97.3) | 98.3 (96.5-99.3) | 100% |
| Richer | − | 94.9 (92.6-96.4) | 97.3 (95.8-98.2) | 98.9 (97.7-99.6) | 100% |
| Richest | − | 96.7 (95.3-97.7) | 98.3 (97.4-98.9) | 99.3 (98.5-99.7) | 100% |
| **Malaria** |  |  |  |  |  |
| **ITNC** | | |  |  |  |
| Poorest | − | 14.7 (6.9-25.9) | 67.7 (42.9-86.3) | 97.7 (85.0-99.9) | 99.9% |
| Poorer | − | 7.6 (3.4-14.1) | 75.7 (52.2-90.9) | 99.6 (97.7-100.0) | 99.9% |
| Middle class | − | 8.9 (3.9-15.8) | 65.7 (39.9-85.9) | 98.5 (89.5-100.0) | 99.4% |
| Richer | − | 8.6 (3.8-16.6) | 53.7 (27.7-78.3) | 95.4 (72.4-99.9) | 95.3% |
| Richest | − | 12.1 (5.6-21.9) | 44.5 (20.2-73.0) | 85.3 (33.8-99.7) | 74.7% |
| **ITNW** | | | |  |  |
| Poorest | − | 11.1 (6.1-18.6) | 71.4 (55.8-83.7) | 99.5 (98.3-99.9) | 100% |
| Poorer | − | 8.5 (4.7-13.7) | 65.1 (48.9-79.2) | 99.3 (97.9-99.9) | 100% |
| Middle class | − | 5.6 (2.9-9.4) | 54.6 (37.9-71.1) | 98.9 (96.9-99.8) | 100% |
| Richer | − | 4.1 (2.2-7.0) | 46.7 (30.7-63.2) | 98.5 (95.4-99.7) | 100% |
| Richest | − | 4.5 (2.4-7.3) | 48.8 (32.6-66.0) | 98.6 (95.9-99.8) | 100% |
| **Environmental health** | |  |  |  |  |
| **Improved water** | |  |  |  |  |
| Poorest | 28.7 (18.5-42.3) | 50.8 (38.7-63.7) | 72.5 (59.1-83.4) | 91.1 (80.6-96.8) | 97.9% |
| Poorer | 42.0 (29.2-57.3) | 65.0 (53.5-76.1) | 82.6 (71.9-90.0) | 94.9 (88.0-98.2) | 100% |
| Middle class | 60.9 (46.9-73.9) | 80.1 (70.7-87.4) | 91.2 (85.1-95.4) | 97.6 (94.4-99.2) | 100% |
| Richer | 76.8 (65.5-85.8) | 89.6 (84.3-93.7) | 95.7 (92.7-97.7) | 98.8 (97.2-99.6) | 100% |
| Richest | 90.0 (83.8-94.3) | 95.9 (93.6-97.5) | 98.4 (97.2-99.2) | 99.6 (99.0-99.9) | 100% |
| **Adequate sanitation** | |  |  |  |  |
| Poorest | 0.4 (0.2-0.7) | 1.6 (0.9-2.7) | 6.5 (3.4-11.1) | 37.5 (17.9-58.8) | 0% |
| Poorer | 3.3 (1.7-5.7) | 12.4 (7.7-18.8) | 37.3 (24.6-51.5) | 82.9 (68.4-92.6) | 72.8% |
| Middle class | 14.4 (8.6-21.5) | 41.2 (30.4-51.8) | 74.5 (63.2-84.4) | 95.9 (91.5-98.6) | 99.9% |
| Richer | 30.7 (20.0-42.0) | 64.9 (54.6-74.4) | 88.5 (82.3-93.5) | 98.4 (96.6-99.4) | 100% |
| Richest | 50.1 (36.9-62.2) | 80.9 (72.7-87.2) | 94.6 (91.0-97.1) | 99.3 (98.4-99.8) | 100% |

Note: ^a^The probability of meeting the target of 80% health service coverage by 2030; CrI: credible interval; ITNC: children under 5 who slept under an insecticide-treated bed net; ITNW: pregnant women who slept under an insecticide-treated bed net.
