# Supplementary material for: Trends and projections of universal health coverage indicators in Ghana, 1995-2030: A national and subnational study
Source: PLoS One. 2019 May 22;14(5):e0209126. doi: 10.1371/journal.pone.0209126 (PMC6530887; doi:10.1371/journal.pone.0209126)
Supplement: S6 Table — (DOCX) [file pone.0209126.s007.docx]

**S6 Table: Quintile specific coverage of treatment services for delivery care and childhood illnesses in Ghana, 1995-2030**

| Indicators | Predicted coverage in year (95% CrI) | | | | | | | Probability^a^ | |  |
| --- | --- | --- | --- | --- | --- | --- | --- | --- | --- | --- |
|  | **1995** | **2005** | | **2015** | | **2030** | |  |  |  |
| Delivery care | | | | |  | |  | |  | |
| INSD |  |  | |  | |  | |  | |  |
| Poorest | 14.7 (10.6-19.4) | 29.2 (22.8-36.1) | | 49.5 (39.9-59.1) | | 78.0 (66.2-86.9) | | 38.8% | |  |
| Poorer | 22.8 (17.1-29.4) | 41.3 (33.4-49.4) | | 62.6 (52.4-71.7) | | 85.7 (76.6-92.0) | | 92.4% | |  |
| Middle class | 35.2 (27.3-44.0) | 56.3 (48.4-63.7) | | 75.4 (67.4-82.0) | | 91.7 (86.2-95.4) | | 100% | |  |
| Richer | 63.1 (54.6-70.9) | 80.3 (74.8-84.9) | | 90.6 (86.7-93.5) | | 97.2 (95.1-98.5) | | 100% | |  |
| Richest | 84.0 (78.3-88.5) | 92.6 (90.2-94.7) | | 96.8 (95.4-97.8) | | 99.1 (98.4-99.5) | | 100% | |  |
| SBA |  |  | |  | |  | |  | |  |
| Poorest | 20.2 (13.4-28.9) | 28.5 (22.2-35.3) | | 38.9 (25.3-53.7) | | 55.3 (26.9-80.9) | | 3.2% | |  |
| Poorer | 23.9 (16.0-33.0) | 42.4 (34.7-51.1) | | 63.2 (49.9-76.4) | | 84.9 (67.0-95.4) | | 77.8% | |  |
| Middle class | 36.1 (25.6-47.2) | 58.4 (50.6-65.9) | | 77.5 (65.7-86.8) | | 92.3 (80.4-98.1) | | 97.7% | |  |
| Richer | 59.6 (47.8-71.0) | 81.8 (76.5-86.3) | | 93.0 (88.6-96.3) | | 98.4 (95.8-99.6) | | 100% | |  |
| Richest | 83.8 (76.6-89.4) | 93.5 (91.2-95.3) | | 97.5 (95.6-98.7) | | 99.3 (98.3-99.8) | | 100% | |  |
| Childhood illness management | | |  | |  | |  | |  | |
| ORT |  |  | |  | |  | |  | |  |
| Poorest | 32.4 (24.1-41.6) | 48.5 (39.3-58.5) | | 64.9 (53.8-75.2) | | 83.2 (71.8-91.6) | | 75.5% | |  |
| Poorer | 37.9 (27.1-48.7) | 54.5 (44.6-63.8) | | 70.2 (59.6-79.2) | | 86.3 (76.1-93.1) | | 91.0% | |  |
| Middle class | 40.4 (30.4-52.1) | 57.1 (47.5-66.7) | | 72.3 (61.0-81.6) | | 87.5 (77.3-93.9) | | 94.3% | |  |
| Richer | 46.7 (34.8-57.5) | 63.3 (53.2-72.2) | | 77.1 (67.4-85.2) | | 90.0 (81.9-95.5) | | 98.9% | |  |
| Richest | 47.0 (35.9-57.6) | 63.5 (53.9-71.8) | | 77.4 (68.7-85.0) | | 90.1 (82.7-95.3) | | 99.3% | |  |
| CPNM |  |  | |  | |  | |  | |  |
| Poorest | 21.3 (13.1-31.8) | 34.3 (25.8-43.7) | | 50.5 (34.2-68.1) | | 71.8 (41.0-92.2) | | 29.8% | |  |
| Poorer | 33.2 (20.7-46.6) | 34.2 (25.0-43.0) | | 35.7 (20.3-54.1) | | 38.5 (12.1-72.6) | | 0.7% | |  |
| Middle class | 40.7 (26.9-55.3) | 50.7 (40.5-61.0) | | 60.4 (42.7-77.4) | | 71.8 (38.7-93.3) | | 31.9% | |  |
| Richer | 44.4 (30.5-59.3) | 49.2 (39.1-59.6) | | 54.0 (36.6-71.6) | | 60.2 (28.1-87.8) | | 10.7% | |  |
| Richest | 54.8 (40.6-68.6) | 52.5 (42.2-61.7) | | 50.1 (31.9-67.2) | | 46.9 (16.1-79.3) | | 2.5% | |  |

Note: ^a^the probability of meeting the target of 80% health service coverage by 2030; CrI: credible interval; INSD: institutional delivery; SBA: skilled birth attendance; ORT: oral rehydration therapy for diarrheal treatment; CPNM: care seeking for pneumonia
