# Supplementary material for: Trends and projections of universal health coverage indicators in Ghana, 1995-2030: A national and subnational study
Source: PLoS One. 2019 May 22;14(5):e0209126. doi: 10.1371/journal.pone.0209126 (PMC6530887; doi:10.1371/journal.pone.0209126)
Supplement: S7 Table — (DOCX) [file pone.0209126.s008.docx]

**S7 Table: Quintile-specific coverage of composite indices for health services in Ghana, 1995-2030**

| **Index** | **Predicted coverage in year (95% CrI)** | | | | |
| --- | --- | --- | --- | --- | --- |
|  | **1995** | **2005** | **2015** | **2030** | **Probability^a^** |
| **Composite prevention index** | |  |  |  |  |
| Poorest | 36.3 (31.6-41.5) | 53.1 (48.3-57.8) | 69.1 (63.9-73.8) | 86.1 (81.5-89.9) | 98.8% |
| Poorer | 45.0 (39.4-50.1) | 61.8 (58.0-66.5) | 76.2 (71.8-80.3) | 89.8 (86.2-92.7) | 100% |
| Middle class | 53.8 (48.8-58.9) | 69.8 (65.9-73.5) | 82.0 (78.7-85.1) | 92.7 (90.0-94.7) | 100% |
| Richer | 63.4 (59.1-68.8) | 78.1 (74.5-81.1) | 87.6 (84.7-89.7) | 95.1 (93.1-96.5) | 100% |
| Richest | 73.9 (69.5-77.8) | 84.8 (82.2-87.2) | 91.7 (89.8-93.3) | 96.8 (95.6-97.7) | 100% |
| Overall | 54.5 (46.6-1.4) | 70.0 (66.1-74.0) | 81.9 (76.3-87.0) | 92.2 (85.4-96.5) | 99.2% |
| **Composite treatment index** | |  |  |  |  |
| Poorest | 24.8 (21.5-28.2) | 36.6 (33.1-40.4) | 50.2 (45.2-55.9) | 70.0 (62.9-76.8) | 0.1% |
| Poorer | 32.3 (28.2-36.6) | 45.5 (41.2-50.2) | 59.4 (54.1-64.4) | 77.1 (70.7-82.7) | 15.2% |
| Middle class | 43.6 (38.9-48.2) | 57.5 (53.3-61.5) | 70.3 (65.9-74.4) | 84.5 (80.1-88.4) | 97.8% |
| Richer | 59.8 (54.8-63.9) | 72.3 (68.4-75.5) | 82.0 (78.6-85.0) | 91.3 (88.5-93.7) | 100% |
| Richest | 74.2 (70.2-77.6) | 83.5 (80.9-85.7) | 89.8 (87.9-91.6) | 95.3 (93.8-96.6) | 100% |
| Overall | 43.2 (36.5-49.8) | 55.4 (50.9-60.0) | 66.8 (59.1-74.0) | 80.3 (67.7-89.4) | 56.3% |
| **Composite coverage index** | |  |  |  |  |
| Poorest | 40.4 (37.5-43.5) | 52.3 (50.1-54.3) | 63.9 (60.3-67.2) | 78.3 (72.8-83.1) | 26.0% |
| Poorer | 47.6 (44.5-50.7) | 58.4 (56.3-60.4) | 68.4 (64.9-71.6) | 80.4 (74.8-84.8) | 58.4% |
| Middle class | 54.4 (51.4-57.3) | 64.2 (62.2-66.0) | 70.3 (69.9-75.8) | 83.2 (78.9-87.1) | 93.6% |
| Richer | 62.8 (59.8-65.6) | 69.8 (67.8-71.5) | 76.0 (73.0-78.7) | 83.4 (78.9-87.3) | 94.1% |
| Richest | 74.4 (72.1-76.6) | 76.0 (74.4-77.5) | 77.5 (75.0-80.3) | 79.6 (74.8-84.5) | 43.4% |
| Overall | 54.7 (52.5-56.6) | 63.3 (62.0-64.6) | 71.1 (69.1-73.3) | 80.7 (77.3-83.9) | 84.2% |

Note: ^a^the probability of meeting the target of 80% health service coverage by 2030; CrI: credible interval
