# Supplementary material for: Trends and projections of universal health coverage indicators in Ghana, 1995-2030: A national and subnational study
Source: PLoS One. 2019 May 22;14(5):e0209126. doi: 10.1371/journal.pone.0209126 (PMC6530887; doi:10.1371/journal.pone.0209126)
Supplement: S8 Table — (DOCX) [file pone.0209126.s009.docx]

**S8 Table: Relative index of inequalities (RII) in health service indicators, 1995-2030**

| **Indicators** | | **RII (95% CrI) (Q5/Q1)^a^** | | | | |
| --- | --- | --- | --- | --- | --- | --- |
|  |  | **1995** | **2005** | **2015** | **2030** |  |
| **Prevention indicators** | | |  |  |  |  |
| **FPS** | | 3.0 (2.0-4.2) | 2.0 (1.4-2.7) | 1.4 (0.6-2.6) | 0.9 (0.2-2.9) |  |
| **ANC4+** | | 2.6 (2.2-3.1) | 1.9 (1.7-2.1) | 1.4 (1.1-.1.6) | 0.8 (0.5-1.2) |  |
| **PNC** | | − | 9.2 (3.6-17.8) | 2.3 (1.0-4.1) | 0.6 (0.0-3.2) |  |
| **EBF** | | 2.2 (1.3-3.4) | 1.3 (0.9-1.9) | 0.9 (0.4-1.7) | 0.5 (0.1-1.7) |  |
| **ITNC^b^** | | − | − | − | − |  |
| **ITNW^b^** | | − | − | − | − |  |
| **BCG** | | 1.4 (1.1-1.7) | 1.2 (1.0-1.4) | 1.0 (0.7-1.4) | 0.8 (0.4-1.6) |  |
| **DPT3** | | 1.8 (1.4-2.4) | 1.3 (1.1-1.6) | 1.0 (0.7-1.4) | 0.6 (0.3-1.2) |  |
| **Polio3** | | 1.8 (1.4-2.3) | 1.3 (1.0-1.5) | 0.9 (0.6-1.2) | 0.5 (0.3-1.0) |  |
| **MSL** | | 1.8 (1.3-2.3) | 1.3 (1.1-1.6) | 1.0 (0.7-1.4) | 0.7 (0.3-1.3) |  |
| **Impwater** | | 4.9 (3.8-6.0) | 2.2 (1.6-2.8) | 1.4 (1.2-1.7) | 1.1 (1.0-1.2) |  |
| **Sanitation** | | 35.4 (15.3-58.3) | 18.2 (11.9-26.1) | 8.9 (3.6-18.3) | 3.8 (0.3-16.2) |  |
| **Ntobacco** | | − | 1.2 (1.0-1.4) | 1.1 (1.0-1.2) | 1.1 (1.0-1.1) |  |
| **Treatment indicators** | | |  |  |  |  |
| **INSD** | 9.0 (5.8-13.0) | | 5.7 (4.3-7.7) | 3.7 (2.1-6.2) | 2.0 (0.6-5.1) |  |
| **SBA** | 8.2 (5.1-12.1) | | 5.5 (4.0-7.4) | 3.8 (2.0-6.4) | 2.4 (0.6-6.7) |  |
| **ORT** | 1.6 (1.2-2.1) | | 1.3 (1.1-1.6) | 1.1 (0.8-1.6) | 0.9 (0.4-1.9) |  |
| **CPNM** | 2.9 (2.3-3.7) | | 2.0 (1.7-2.4) | 1.5 (1.0-2.0) | 0.9 (0.5-1.6) |  |

Note: ^a^RII, relative index of inequality; Q5 indicates the richest quintile, and Q1 indicates the poorest quintile; CrI: credible interval; ANC4+: at least four antenatal care visits; PNC: post-natal care of mother; BCG: BCG immunization; DPT3: three doses of DPT immunization; Polio3: three doses of polio immunization; MSL: measles vaccination; EBF: exclusive breastfeeding; FPS: family planning needs satisfied; NTobacco: non-use of tobacco; ImpWater: improved water; Sanitation: adequate sanitation; INSD: institutional delivery; SBA: skilled birth attendance; ORT: oral rehydration therapy for diarrheal treatment; CPNM: care seeking for pneumonia; ITNC: children under 5 who slept under an insecticide-treated bed net; ITNW: pregnant women who slept under an insecticide-treated bed net
