# Supplementary material for: Trends and projections of universal health coverage indicators in Ghana, 1995-2030: A national and subnational study
Source: PLoS One. 2019 May 22;14(5):e0209126. doi: 10.1371/journal.pone.0209126 (PMC6530887; doi:10.1371/journal.pone.0209126)
Supplement: S9 Table — (DOCX) [file pone.0209126.s010.docx]

**S9 Table: Overall prevention and treatment service coverage at the subnational level in Ghana, 1995-2030**

| **Index** | **Predicted coverage in year (95% CrI)** | | | | | **Probability^a^** |
| --- | --- | --- | --- | --- | --- | --- |
|  | **1995** | | **2005** | **2015** | **2030** |  |
| **Composite prevention index** | |  | |  |  |  |
| Ashanti | 58.5 (54.5-62.6) | | 73.9 (70.9-76.9) | 85.1 (82.7-87.2) | 94.2 (92.7-95.4) | 100% |
| Brong-Ahafo | 58.0 (53.9-62.1) | | 73.5 (70.4-76.5) | 84.8 (82.2-87.0) | 94.1 (92.5-95.3) | 100% |
| Central | 51.9 (47.7-56.3) | | 68.5 (64.8-71.8) | 81.4 (78.5-84.0) | 92.5 (90.7-94.1) | 100% |
| Eastern | 51.3 (46.9-55.6) | | 67.9 (64.3-71.4) | 81.0 (77.9-83.6) | 92.4 (90.2-93.9) | 100% |
| Greater Accra | 65.5 (61.5-69.5) | | 79.2 (76.5-81.9) | 88.5 (86.5-90.2) | 95.6 (94.5-96.6) | 100% |
| Northern | 38.3 (34.2-42.4) | | 55.5 (51.5-59.5) | 71.5 (67.7-75.2) | 87.7 (84.8-90.2) | 100% |
| Upper East | 54.2 (49.8-58.5) | | 70.4 (66.8-73.9) | 82.7 (79.8-85.3) | 93.2 (91.3-94.7) | 100% |
| Upper West | 54.8 (50.5-58.9) | | 70.9 (67.6-74.2) | 83.0 (80.4-85.5) | 93.3 (91.4-94.7) | 100% |
| Volta | 49.7 (45.3-53.9) | | 66.5 (62.8-70.0) | 80.0 (76.9-82.5) | 91.9 (89.9-95.3) | 100% |
| Western | 51.9 (47.9-56.3) | | 68.5 (65.1-71.8) | 81.4 (78.4-84.1) | 92.5 (90.6-94.1) | 100% |
| **Composite treatment index** | |  | |  |  |  |
| Ashanti | 52.6 (43.2-61.8) | | 64.5 (58.4-69.8) | 74.6 (64.9-82.6) | 85.3 (70.9-94.2) | 83.1% |
| Brong-Ahafo | 47.8 (38.4-56.7) | | 61.3 (55.8-67.3) | 73.2 (63.5-81.6) | 85.5 (70.9-94.5) | 85.8% |
| Central | 35.2 (27.4-43.6) | | 52.3 (46.0-58.2) | 68.7 (57.6-78.1) | 85.6 (70.5-94.4) | 85.5% |
| Eastern | 45.7 (37.2-54.8) | | 56.8 (50.5-62.7) | 67.2 (56.4-77.1) | 79.2 (61.5-91.4) | 52.6% |
| Great Accra | 66.9 (58.4-74.7) | | 70.0 (64.4-75.0) | 72.8 (63.4-80.8) | 76.1 (58.9-89.0) | 34.9% |
| Northern | 24.1 (18.0-31.2) | | 33.2 (27.7-39.0) | 43.9 (33.2-55.1) | 60.1 (38.5-79.0) | 1.8% |
| Upper East | 33.1 (25.2-41.6) | | 58.4 (52.1-64.6) | 79.8 (71.7-86.4) | 94.6 (88.6-98.0) | 99.8% |
| Upper West | 31.8 (24.1-39.8) | | 50.2 (44.0-55.9) | 68.4 (57.9-77.7) | 86.6 (72.7-94.9) | 88.8% |
| Volta | 33.2 (25.4-41.6) | | 47.6 (41.5-53.6) | 62.4 (50.9-72.3) | 79.6 (61.1-91.4) | 54.5% |
| Western | 37.5 (29.6-45.8) | | 57.5 (51.2-63.4) | 75.1 (66.3-82.7) | 90.5 (81.0-96.2) | 98.1% |

Note: ^a^the probability of meeting the target of 80% health service coverage by 2030; CrI: credible interval
