# Supplementary material for: Trends and projections of universal health coverage indicators in Ghana, 1995-2030: A national and subnational study
Source: PLoS One. 2019 May 22;14(5):e0209126. doi: 10.1371/journal.pone.0209126 (PMC6530887; doi:10.1371/journal.pone.0209126)
Supplement: S10 Table — (DOCX) [file pone.0209126.s011.docx]

**S10 Table: Impoverishment at the subnational level in Ghana, 1995-2030**

| **Country/Region** | **Impoverishment^a^ (95% CI)** | | | | **Probablity^b^** |
| --- | --- | --- | --- | --- | --- |
|  | **1995** | **2005** | **2015** | **2030** |  |
| Ashanti | 2.1 (1.2-3.2) | 1.1 (0.6-1.6) | 0.5 (0.3-0.8) | 0.2 (0.1-0.4) | 100% |
| Brong-Ahafo | 3.0 (1.9-4.6) | 1.5 (1.0-2.3) | 0.8 (0.5-1.2) | 0.3 (0.1-0.5) | 100% |
| Central | 0.4 (0.3-0.7) | 0.2 (0.1-0.3) | 0.1 (0.1-0.2) | 0.0 (0.0-0.1) | 100% |
| Eastern | 2.0 (1.2-3.1) | 1.0 (0.6-1.5) | 0.5 (0.3-0.8) | 0.2 (0.1-0.3) | 100% |
| Great Accra | 2.3 (1.4-3.7) | 1.2 (0.7-1.8) | 0.6 (0.3-1.0) | 0.2 (0.1-0.4) | 100% |
| Northern | 2.6 (1.6-4.1) | 1.3 (0.8-2.0) | 0.6 (0.4-1.0) | 0.2 (0.1-0.4) | 100% |
| Upper East | 3.2 (1.9-5.0) | 1.6 (1.0-2.6) | 0.8 (0.5-1.4) | 0.3 (0.1-0.6) | 99.9% |
| Upper West | 1.4 (0.9-2.2) | 0.7 (0.4-1.1) | 0.4 (0.2-0.6) | 0.1 (0.1-0.2) | 100% |
| Volta | 1.3 (0.7-2.1) | 0.6 (0.4-1.0) | 0.3 (0.2-0.5) | 0.1 (0.0-0.2) | 100% |
| Western | 1.1 (0.6-1.7) | 0.5 (0.3-0.8) | 0.3 (0.2-0.5) | 0.1 (0.0-0.2) | 100% |

Note: ^a^impoverishment was calculated based on a food shared based poverty line; ^b^The probability of achieving 100% financial risk protection; CrI: credible interval
