# Supplementary material for: Trends and projections of universal health coverage indicators in Ghana, 1995-2030: A national and subnational study
Source: PLoS One. 2019 May 22;14(5):e0209126. doi: 10.1371/journal.pone.0209126 (PMC6530887; doi:10.1371/journal.pone.0209126)
Supplement: S11 Table — (DOCX) [file pone.0209126.s012.docx]

**S11 Table: Survey characteristics^a^**

| **Survey** | **Year** | **Sample design** | **Number of households** | **Household response rate** |
| --- | --- | --- | --- | --- |
| Demographic Health and Survey | 1993 | Two-stage stratified sampling | 6,161 | 98.4% |
|  | 1998 | Two-stage stratified sampling | 6,055 | 99.1% |
|  | 2003 | Two-stage stratified sampling | 6,333 | 98.7% |
|  | 2008 | Two-stage stratified sampling | 11,913 | 98.9% |
|  | 2014 | Two-stage stratified sampling | 12,010 | 98.5% |
| Ghana Living Standard Survey | 1991-1992 | Multi-stage sampling | 4,565 | 99.7% |
|  | 1998-1999 | Two-stage stratified sampling | 6,000 | 99.7% |
|  | 2005-2006 | Two-stage stratified random sampling | 8,700 | 99.9% |
|  | 2012-2013 | Two-stage stratified sampling | 18,000 | 93.2% |

Note: ^a^all information taken from survey reports^4-12^
