# Supplementary material for: Trends and projections of universal health coverage indicators in Ghana, 1995-2030: A national and subnational study
Source: PLoS One. 2019 May 22;14(5):e0209126. doi: 10.1371/journal.pone.0209126 (PMC6530887; doi:10.1371/journal.pone.0209126)
Supplement: S2 Fig — (PDF) [file pone.0209126.s014.pdf]

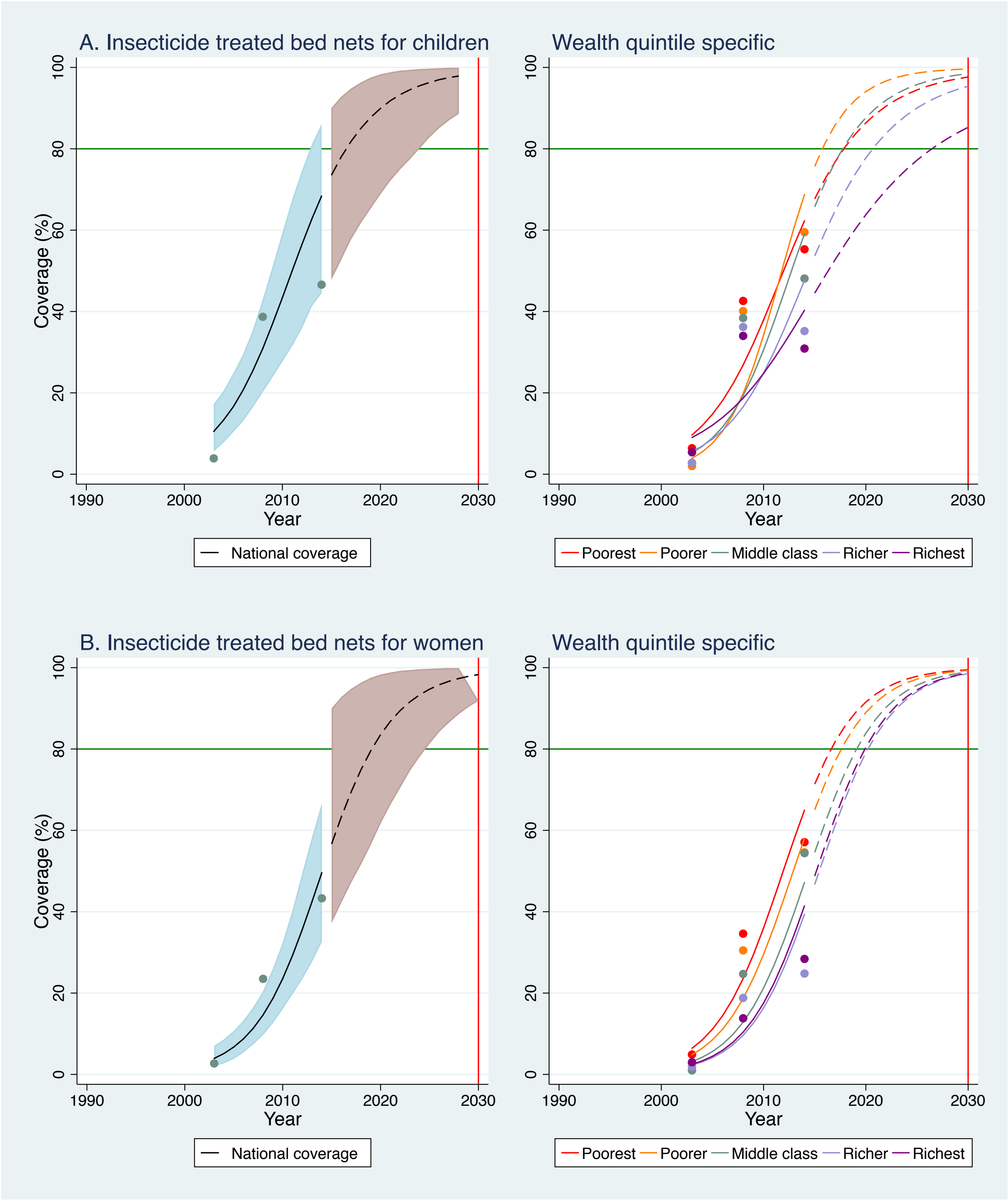

S2 Fig: Trends and projections of the coverage of insecticide treated bed nets for children under five and pregnant women in Ghana, 2003-2030

Note: (A) Coverage of insecticide treated bed nets for children under five (B) Coverage of insecticide treated bed nets for pregnant women

The figures on the left panel represent national coverage, and the figures on the right panel show coverage across wealth quintiles. The dots indicate observed estimates from raw data. The lines show the posterior mean estimates (dashed line for projected values for future); the shaded area shows the 95% credible intervals
